# The mir‐200 family regulates key pathogenic events in ascending aortas of individuals with bicuspid aortic valves

**DOI:** 10.1111/joim.12833

**Published:** 2018-10-02

**Authors:** S. Maleki, K. A. Cottrill, F.‐A. Poujade, A. Bhattachariya, O. Bergman, J. R. Gådin, N. Simon, K. Lundströmer, A. Franco‐Cereceda, H. M. Björck, S. Y. Chan, P. Eriksson

**Affiliations:** ^1^ Department of Medicine Cardiovascular Medicine Unit Center for Molecular Medicine Karolinska Institutet Stockholm Sweden; ^2^ Karolinska University Hospital Solna Sweden; ^3^ Division of Cardiology Department of Medicine Pittsburgh Heart, Lung, Blood, and Vascular Medicine Institute University of Pittsburgh School of Medicine University of Pittsburgh Medical Center Pittsburgh PA USA; ^4^ Department of Molecular Medicine and Surgery Cardiothoracic Surgery Unit Karolinska Institutet Stockholm Sweden

**Keywords:** aortic aneurysm, bicuspid aortic valve, microRNA

## Abstract

**Background:**

An individual with a bicuspid aortic valve (BAV) runs a substantially higher risk of developing aneurysm in the ascending aorta compared to the normal population with tricuspid aortic valves (TAV). Aneurysm formation in patients with BAV and TAV is known to be distinct at the molecular level but the underlying mechanisms are undefined. Here, we investigated the still incompletely described role of microRNAs (miRNAs), important post‐transcriptional regulators of gene expression, in such aortic disease of patients with BAV as compared with TAV.

**Methods and Results:**

Using a system biology approach, based on data obtained from proteomic analysis of non‐dilated aortas from BAV and TAV patients, we constructed a gene‐interaction network of regulatory microRNAs associated with the observed differential protein signature. The miR‐200 family was the highest ranked miRNA, hence potentially having the strongest effect on the signalling network associated with BAV. Further, qRT‐PCR and ChIP analyses showed lower expression of miR‐200c, higher expression of miR‐200 target genes, ZEB1/ZEB2 transcription factors, and higher chromatin occupancy of the miR‐200c promoter by ZEB1/ZEB2 in BAV patients, indicating a miR‐200c/ZEBs negative feedback loop and induction of endothelial/epithelial mesenchymal transition (EndMT/EMT).

**Conclusion:**

We propose that a miR‐200‐dependent process of EndMT/EMT is a plausible biological mechanism rendering the BAV ascending aorta more prone to aneurysm development. Although initially supported by a miR‐200c/ZEB feedback loop, this process is most probably advanced by cooperation of other miRNAs.

## Introduction

An individual with a bicuspid aortic valve (BAV) runs higher risk of developing aortic complications such as valve stenosis or regurgitation, as well as aneurysm in ascending aorta and dissection, compared with individuals with a normal tricuspid aortic valve (TAV). It has been proposed that inherent changes, as a consequence of genetic mutation or altered flow pattern, cause the increased aneurysm susceptibility in the BAV population 1, 2. At the molecular level, the events leading to aneurysm formation are different in patients with BAV and TAV 3, 4, 5.

MicroRNAs (miRNAs) are short noncoding RNAs of about 22 nucleotides that bind to mRNAs on the complementary site, and control gene expression by translation inhibition or target degradation. MicroRNA regulation has emerged as a fundamental mechanism for modulation of normal biological processes as well as dysregulations leading to pathological conditions. The role of microRNAs in different types of aneurysms has been reviewed recently 6, 7, and the role of circulating microRNAs in BAV‐associated aneurysm in comparison with TAV has been discussed 8. Also, lately, a comparative study analysing microRNA expression profile differences between BAV and TAV aneurysmal aorta has been reported 9. However, the differences in the microRNA expression in nondilated ascending aortas of BAV and TAV patients have not been reported so far.

Using a combination of proteomics/genome analysis/microscopy, we have previously shown a molecular signature of EndMT/EMT in the intima and possibly in the media of the BAV ascending aorta prior to dilatation 5, 10. EndMT/EMT is a complex molecular and cellular re‐programming that is activated during normal physiological processes such as embryonic development and wound healing, as well as in several pathological circumstances such as cancer and tissue fibrosis. EndMT/EMT is divided into three subtypes representing distinct biological processes, resulting in production of different cell types, that is mesenchymal cells in the case of type I activated during embryonic development, fibroblasts in the type II involved in fibrosis and wound healing, and invasive tumour cells in the type III associated with cancer. All three types involve the loss of endothelial/epithelial cells features and acquisition of mesenchymal traits, that is loss of cell apical and basal polarity and junctions, and gain of enhanced motility and invasiveness 11, 12. In order to investigate the possible regulatory role of microRNAs to the observed EndMT/EMT phenotype in BAV, we applied an *in silico* approach 13 to the previously identified differentially expressed proteins between nondilated and aneurysmal aortas, respectively, from BAV and TAV patients, to construct a network of regulatory microRNAs. This procedure identified the miR‐200 family as key regulator of the ongoing biological process that differs between BAV and TAV nondilated aorta. We further confirm the activation of an EndMT/EMT‐like mechanism in the BAV nondilated aortic wall by studying the differential expression of key modulators of this process controlled by mir‐200 family members. We discovered that mir‐200c and its direct targets ZEB1/2 were differentially expressed between BAV and TAV aorta prior to dilation. Moreover, we show that the EndMT/EMT‐like process is, at least partly, due to the establishment of the miR‐200c/ZEBs negative feedback loop that has also been characterized in different types of cancer.

## Material and methods

### Study subjects

Tissue biopsies were acquired from patients undergoing elective open‐heart surgery at the Karolinska University Hospital, Sweden, for aortic valve and/or ascending aortic disease. Patients were classified based on aortic valve cuspidity and dilatation (aortic diameters of >45 mm and <40 mm were classified as dilated (D) and nondilated (ND), respectively). ND patients were operated on due to valve dysfunction. Patients with syndromic aortic pathologies, dissection and/or significant coronary artery disease were excluded from the study. Aortic biopsies were obtained from the anterior part of the ascending aorta, a few cm above the aortic valve, and from the proximal portion of the internal thoracic artery. The intima–media of the aortic wall was separated from the adventitia; only the intima–media part of the aorta was used for our study. In total, 66 BAV (45 BAV‐ND, 21 BAV‐D) and 63 TAV (45 TAV‐ND, 18 TAV‐D) were included. Characteristics of patients are shown in Table S1. The study was approved by the Human Research Ethics Committee at Karolinska Institutet (application number 2012/1633‐31/4), Stockholm, Sweden; written informed consent was obtained from all the patients according to the Declaration of Helsinki, and methods were carried out in accordance with relevant guidelines.

### Network analysis

**Figure 1:**
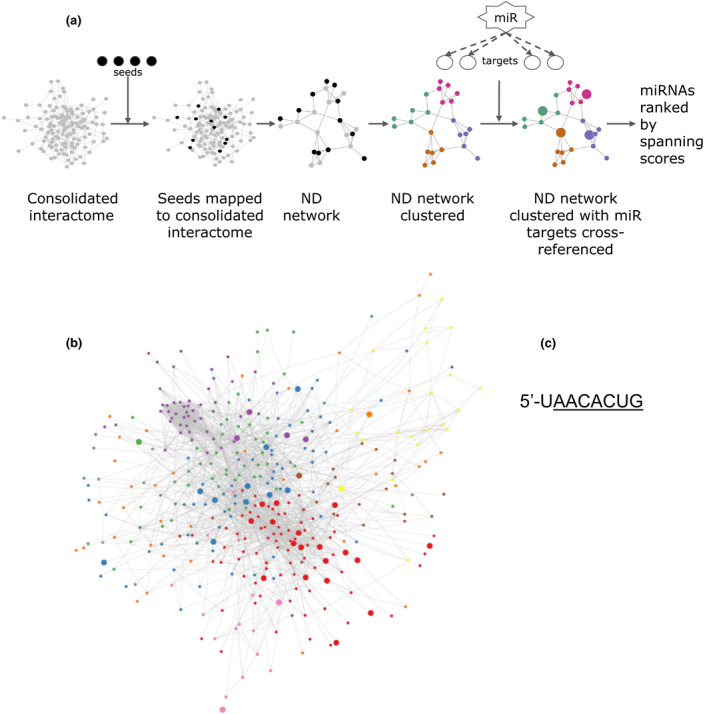
Computational network analysis (a) Flow chart of the network analysis pipeline; (b) cluster modules of highly interacting groups of genes in the nondilated (ND) network. Different colours in the network represent different cluster modules (nonlabelled). The network was clustered by a spectral partition‐based clustering algorithm using ReactomeFI in Cytoscape. miRNA targets were cross‐referenced to the network and regulatory miRNAs were identified and ranked according to calculated spanning scores (see Materials and methods for details). Module assignments/labels and spanning scores are provided in Supporting Information. Target genes, visualized as larger nodes in the ND network, of the top‐ranked miRNA‐200 family are presented. The shared sequence for members of the miR‐200 family (functional group 200b, c and 429), with seed sequence underlined, was 5′UAACACUG.

We applied a network analysis using the methods designed by Chan *et al*. 13. We used a consolidated interactome that consisted of functional molecular associations curated from a variety of human genes and molecular interaction databases 13. Our previous study 5 identified 276 proteins that were differentially expressed between nondilated BAV and TAV aorta. We used genes that expressed these proteins as seeds to build a nondilated (ND) network from the consolidated interactome. The ND network consisted of seed genes that were mapped to the consolidated interactome, as well as their direct, first‐degree interactors. The ND network was then clustered using a spectral partition‐based algorithm, as implemented in ReactomeFI 14 to find modules of highly interacting groups of genes. Finally, microRNA targets were cross‐referenced to our clustered ND network. MicroRNA targets were downloaded from TargetScan 7.1 15. The total number of available microRNA in TargetScan is 153. For each microRNA, a ‘microRNA spanning score’ was calculated using methods described by Chan *et al*. 13 to detect global regulatory effects. Fig. 1a shows the flow chart for the network analysis. We also performed a functional pathway enrichment analysis of all genes in the ND network, using hypergeometric tests based on the hallmark gene sets curated by MSigDB 16. The network analysis was further applied to proteins differentially expressed between aneurysmal BAV and TAV aorta (*N* = 805).

### *In situ* hybridization (ISH)

*In situ* hybridization (ISH) was performed essentially as described previously 17 in cooperation with Bioneer (Bioneer A/S, Hørsholm, Denmark). In brief, ascending aortic biopsies from BAV and TAV patients were fixed in standard formalin, paraffin‐embedded and cut into 5‐μm‐thick sections. Following de‐paraffinization, double‐digoxigenin‐labelled locked nucleic acid (LNA) probes for miR‐200c‐3p (TCCATCATTACCCGGCAGTATTA, predicted RNA Tm: 84 °C) and scramble (TGTAACACGTCTATACGCCCA, predicted RNA Tm: 87 °C) were obtained from Exiqon (Exiqon A/S, Vedbæk, Denmark). The probes were incubated at 10–40 nmol L^−1^ in Exiqon ISH buffer (Exiqon) at 55–60 °C. The probes were detected with alkaline phosphatase‐conjugated anti‐DIG antibodies (Merck, Kenilworth, NJ, USA) and incubated in 4‐nitroblue tetrazolium (NBT) and 5‐bromo‐4‐chloro‐3′‐indolylphosphate (BCIP) substrate (Merck) for 2 h. The sections were counterstained with Nuclear Fast Red (Vector Laboratories, Burlingame, CA, USA) and finally dehydrated in ethanol dilutions and mounted.

### RNA isolation and qRT‐PCR

Total RNA was isolated from endothelial cells and aortic tissue using miRNeasy mini kit (Qiagen, Hilden, Germany) and quantified using NanoDrop ND‐1000 (NanoDrop Technologies, Wilmington, DE, USA) according to the manufacturer's instructions. For mRNA, cDNA was synthesized using 0.5 μg of RNA from each sample with random primers and Superscript II (Invitrogen, Carlsbad, CA, USA). For miRNA, cDNA was synthesized using TaqMan™ MicroRNA Reverse Transcription Kit #4366596 (Thermo Fisher Scientific, Waltham, MA, USA, 4366596), using specific primers for each microRNA.

The relative expression of mRNA was analysed by real‐time qPCR (StepOnePlus qPCR cycler, Applied Biosystems, Foster City, CA, USA) using TaqManTM Gene Expression Master Mix (Thermo Fisher Scientific, #4369510) with TaqMan Gene Expression Assays (Thermo Fisher Scientific, ZEB1, #Hs01566408_m1; ZEB2, #Hs00207691_m1; PPIA, #Hs04194521_s1; SNAI1, Hs00195591_m1). For miRNA, TaqMan Universal Master Mix II with UNG (Thermo Fisher Scientific #4440044) was used with TaqManTM MicroRNA Assays (Thermo Fisher Scientific, #4427975, Assay ID: 002300; hsa‐miR‐200c, Assay ID: 001024; hsamiR‐429, Assay ID: 002251; hsa‐miR‐200b, Assay ID: 001973; U6 snRNA). The experiments were carried according to the manufacturer's instructions. The number of patients for each qRT‐PCR measurement is given in each figure legend, respectively.

The expression of miR‐200 family members in endothelial cells was quantified using Human Transcriptome Array 2.0 (Affymetrix), as described previously 18, in accordance with manufacturer's instructions. In total, *n* = 6 BAV and *n* = 7 TAV patients were included.

### Immunohistochemistry (IHC)

Immunohistochemistry was performed on de‐paraffinized aortic tissue sections treated with DIVA solution (Biocare Medical, Concord, CA, USA), as described previously 5. The expression of ZEB2 was quantified in *n* = 10 BAV‐ND patients, and *n* = 11 TAV‐ND patients, using mouse monoclonal anti‐ZEB2 (Santa Cruz, Heidelberg, Germany, sc‐271984). Quantification of per cent area positive staining was performed using Image‐Pro^®^ Premier version 9.1 Software (Media Cybernetics^®^, Silver Spring, MD, USA).

### Western blot analysis

Protein expression of ZEB1 was analysed by Western blot using protein lysates prepared by tissue homogenization in RIPA buffer (Thermo Fisher Scientific). Twenty micrograms of protein was re‐suspended in Laemmli sample buffer (BioRad, Hercules, CA, USA) containing 20% DTT and run on SDS‐PAGE using Mini‐PROFEAN TGX 4–20% protein gels (BioRad 456‐1093) for 40 min at 140V. Proteins transfer was performed with Tran‐Blot turbo system (Bio‐Rad 170‐4156) to nitrocellulose membranes (BioRad) according to standard protocols, followed by blocking in 5% dry‐milk in TTBS. The blots were incubated with primary anti Zeb 1 (goat, ThermoFisher, Stockholm, Sweden, PA5‐19078) and anti‐beta‐Actin (mouse, Sigma‐Aldrich A1978, Stockholm, Sweden), 48 h for anti‐Zeb1 and 1 h for beta‐actin, followed by 1 h incubation with horseradish peroxidase–labelled secondary antibodies (rabbit anti‐goat HRP, Dako P0449, goat anti‐mouse HRP BioRab 170‐6516). Enhanced chemiluminescence Western blot detection reagent (Amersham, GE Healthcare, Uppsala, Sweden) was used for detection.

### Transmission electron microscopy (TEM)

Transmission electron microscopy was done as described previously 5. Samples of ascending aorta were fixed in 2% glutaraldehyde +1% paraformaldehyde in 0.1 mol L^−1^ phosphate buffer, pH 7.4 at room temperature and stored at 4 °C. Postfixation was carried out in 2% osmium tetroxide 0.1 mol L^−1^ phosphate buffer, pH 7.4 at 4 °C. Ultrathin sections of about 50–60 nm were contrasted with uranyl acetate followed by lead citrate and viewed in a Tecnai 12 Spirit Bio TWIN transmission electron microscope (FEI company, Eindhoven, the Netherlands) at 100 kV.

### Cell culture and LNA protocol

Human aortic endothelial cells (HAEC) were isolated from aneurysmal aorta of BAV and TAV patients, as described previously 18, and cultured in Endothelial Cell Basal media with growth supplements, including 10% foetal calf serum and penicillin/streptomycin (PromoCell, Heidelberg, Germany) with 5% CO^2^ at 37 °C. Primary aortic cells were used in passage 3–4. Human EA.hy926 cells 19 were cultured in Dulbecco's modified Eagle's high glucose medium (DMEM) supplemented with 10% foetal calf serum and penicillin/streptomycin at 37 °C with 5% CO^2^. EA.hy926 cells were transfected with commercially available LNA™ Oligonucleotides (Exiqon, Vedbaek, Denmark, #450012) for hsa‐miR‐200 family or negative control (Exiqon, #199006‐001) using Lipofectamine RNAiMAX Transfection Reagent (Thermofisher, #13778075). Expression levels of ZEB1 and SNAI1 were measured to establish the effect of microRNA‐200 family on the target transcription factors.

### Chromatin immunoprecipitation–quantitative PCR (ChIP‐qRT‐PCR)

Approximately 50 mg of frozen tissue sample is cut into small pieces and protein/DNA complexes were crosslinked by adding formaldehyde directly to the PBS to a final concentration of 1% for 10 min. After quenching the crosslinking reaction with 0.65 mol L^−1^ glycine, chromatin was extracted from the cell nuclei according to the manufacturer's instructions (Chromatrap, UK). Briefly, the crosslinked tissue is homogenized using TissueLyser Bead Homogenizer (Qiagen) and subsequently sonicated using the Diagenode Bioruptor (Liège, Belgium) at high settings (10 min, 30 s on/off) and run on 1.5% agarose gel in 1xTBE to check the shearing efficiency (100–500 bp fragments). A total of 100 μL of sheared chromatin was used for slurry preparation and 10 μL was set aside as input. Next, 4 μg of anti‐Zeb1 (#Abcam, Cambridge, UK, ab87280), antit‐Zeb2 (Santa Cruz, sc‐271984) and anti‐IgG (AbCam, ab2410) was added to the chromatin and incubated overnight in a rocking platform. Samples were then de‐crosslinked at 65 °C overnight and proteins were subsequently degraded for 1 h by incubating with Proteinase K at 37 °C. After adding Proteinase K Stop solution, DNA was purified according to manufacturer's instructions (Chromatrap, UK, #500235) and real‐time qPCR (StepOnePlus qPCR cycler, Applied Biosystems) was performed using TaqMan™ Gene Expression Master Mix (Thermo Fisher Scientific, #4369510). ChIP primer sets were designed for (−480 to −210, and +872 to +995) and the known ZEB binding region. Sequences of the promoter‐specific primers and for the control region used are available in Table S2. Calculation of promotor occupancy and its significance was performed using the Percent Input Method 20.

### Statistical methods

Values are presented as mean ± standard error of the mean (SEM) unless otherwise stated. Correlation between the expression of different miR‐200 family members was calculated using Pearson correlation analysis (R). Differential expression of miR‐200 family members was calculated using linear regression analysis (R), including age, aortic valve disease (stenosis and regurgitation) and ascending aortic diameter as covariates. Differential expression of miR‐200 targets was calculated using Mann–Whitney *U*‐test (GraphPad Prism 5, La jolla, CA, USA). Hallmark analysis of nondilated network genes was calculated using hypergeometric test (R). Differences in protein expression were calculated using student's *t*‐test (GraphPad Prism 5). A *P*‐value of *P* < 0.05 was considered statistically significant, unless otherwise stated. The number of patients included in each analysis is given in each figure caption, respectively. The total number of patients included in the study was 66 BAV and 63 TAV.

## Results

### MicroRNA network analysis identified the miR‐200 family as a potential key regulator of molecular differences between nondilated BAV and TAV aorta

**Figure 2:**
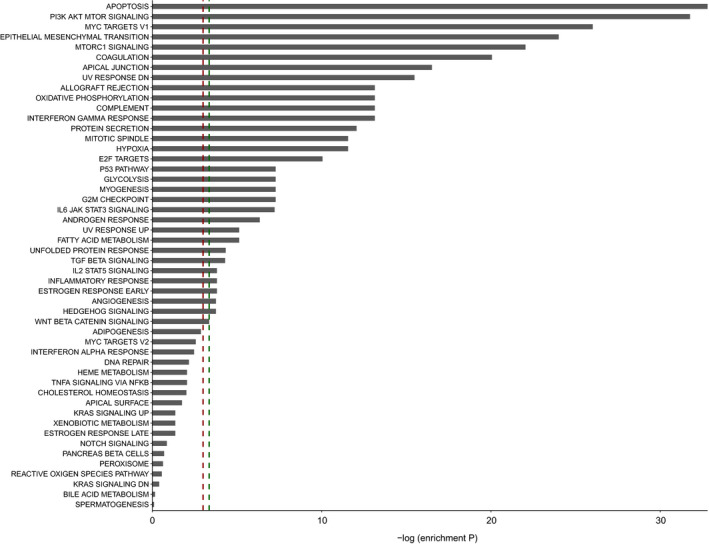
Hallmark analysis of nondilated network genes. In total, *n* = 375 genes were included in the enrichment analysis. ‐logP indicates the enrichment *P*‐value (hypergeometric test). Red dashed line indicates *P*‐value 0.05; green dashed line indicates FDR‐adjusted *P*‐value 0.05. Hallmark gene sets are curated by the Molecular Signatures Database (MSigDB).

To identify possible regulatory miRNAs in mechanisms that predisposes BAV patients to higher incidence of ascending aortic aneurysm, we combined our large‐scale proteomics data from nondilated BAV and TAV aortic tissue 5 with an *in silico* network analysis approach previously described (Fig. 1a) 13. A consolidated interactome, consisting of functional molecular associations curated from a variety of human genes and molecular interaction databases, was used as base in the *in silico* network analysis. Proteins previously identified as differentially expressed between nondilated BAV and TAV aorta were used as seeds (*N* = 276 5). To construct a network specific for the BAV/TAV nondilated (ND) aorta, seeds were mapped to the consolidated interactome. Then, first‐degree interactors of seeds were identified based on the interactome, resulting in a ND network including *n* = 375 genes and *n* = 2210 interactions. In order to find the functional relevance of ND aortic network genes, Hallmark analysis was performed. This revealed that ND network genes were enriched for biological processes such as Apoptosis, PI3K/AKT/mTOR signalling, MYC Targets V1, mTORC1 and EMT (full list in Fig. 2). The PI3K/AKT/mTOR pathway is altered in cancer and activated in autophagy and for overcoming apoptosis in cancer cells 21. Of note, mTORC1 is the master regulator of autophagy, and electron microscopic studies identified authophagosome‐like structure adjacent to endothelial cell membrane of BAV‐ND specimen but not TAV‐ND (Fig. S1). Moreover, MYC targets V1 group of proteins are specifically associated with cancer cells with a fast rate of cell division 22, and MYC is an oncogene, uncontrolled expression of which is associated with a wide range of human cancer. MYC also regulates a number of cell functions such as proliferation, differentiation and apoptosis, all of which are activated during tumorigenesis 23. This fits well with EndMT/EMT molecular phenotype of the nondilated BAV aorta we described previously.

Then, by the use of a spectral clustering algorithm 14, eight modules of highly interacting groups of genes (cluster modules) were identified (Fig. 1b), to which miRNA targets were cross‐referenced (see Fig. 1a). Different colours in the network represent different cluster modules. To detect potential global regulatory miRNAs, a miRNA spanning score was calculated for each miRNA. Of note, the spanning score combines the hypergeometric analysis and the cluster‐based analysis into a single score and reveals the influence of a given miRNA to modulate a diverse range of processes within the network. The miRNAs were then ranked by their calculated spanning score, identifying miR‐200bc/429/548a as the major regulating microRNA in the BAV‐ND network (Table S3, including the totally 153 available microRNAs in TargetScan, and their ranking). MiR‐200bc/429/548a had 44 predicted targets in the network, spread over seven out of the in total eight clusters. This suggests, with high likelihood, that this miRNA is a major regulator, influencing ongoing biological processes that differ between BAV and TAV nondilated ascending aortas. Of note, the miR‐200 family contains five members and forms two different clusters based on their genomic location; cluster I that contains hsa‐miR‐200a, hsa‐miR‐200b and hsa‐miR‐429 and is located on human chromosome 1, and cluster II that contains hsa‐miR‐141 and hsa‐miR‐200c and is located on chromosome 12. Based on their similarity of seed sequences, however, the mir‐200 family members can also be divided into two functional groups: mir‐200b/mir‐200c/miR‐429 and miR‐200a/miR‐141. The two functional groups only differ by one nucleotide in the seed sequence 24. The top‐ranked miRNA, miR‐200bc/429/548a subgroup, will hereafter be referred to as miR‐200 family.

The miR‐200 family has an established role in EndMT/EMT and high association with human cancers 25, 26, 27. However, in order to find the possible involvement of other top‐ranked miRNAs, either in EndMT/EMT or related biological processes, such as cancers, we selected the ten top‐ranked microRNAs and performed a detailed literature search of additional microRNAs. This analysis showed that all ten ranked miRNAs were functionally involved in either promoting or suppressing EndMT/EMT or cancers (Table S4A and references therein). Interestingly, the involvement of mir‐133 28 and mir‐23 29 in EndMT of the endocardium, which is important for proper aortic valve development, has been reported. Furthermore, mir‐29 has a regulatory role in the development of thoracic and abdominal aortic aneurysms 7.

Lastly, we applied the same *in silico* network analysis approach using proteins differentially expressed between dilated BAV and TAV aortas (*n* = 805) 5 to investigate the possible regulatory role of miRNAs in the aneurysmal state (network in Fig. S2). This analysis showed that the miR‐200 family shifted to the third top‐ranked microRNAs (Table S2). However, literature search indicated that all other top nine microRNAs could take part in induction of EndMT/EMT (Table S4B and references therein). Pathway analysis of genes included in the extended network of dilated samples ranked MYC targets V1, apical junction, epithelial mesenchymal transition, mitotic spindle and PI3K/AKT/mTOR signalling as the top five pathways (Fig. S3). Association of mir‐181 and mir‐145 with thoracic aortic aneurysm has also been shown 7.

### Endothelial cells are the main source of miR‐200c expression

**Figure 3:**
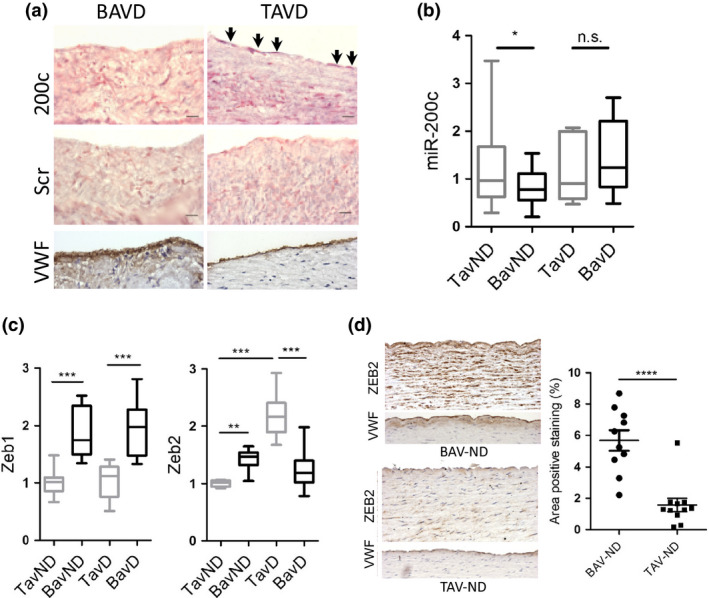
Expression of miR‐200c and its targets ZEB1/ZEB2 in BAV and TAV aortas. (a) *In situ* hybridization of mir‐200c probe to wax‐embedded sections of BAV and TAV dilated aortas. VWF staining shows the location of endothelium. Scale bar = 50 μm, *N* = 4 patients/group. Scr: Scramble control. (b) Comparative qRT‐PCR measurements of miR‐200c and (c) ZEB1 and ZEB2 in BAV and TAV aortic intima–media, dilated (d) and nondilated (ND) aortas. *N* = 21 BAV‐ND, *N* = 12 BAV‐D, *N* = 20 TAV‐ND and *N* = 11 TAV‐D for miR‐200c, and *N* = 12 BAV‐ND, *N* = 12 BAV‐D, *N* = 11 TAV‐ND and *N* = 9 TAV‐D for ZEB1 and ZEB2. *P*‐values are corrected for age, aortic valve disease (stenosis and regurgitation) and aortic diameter (Linear regression). (d) ZEB2 staining in BAV and TAV nondilated aortas. VWF staining shows the location of endothelium. Scale bar = 50 μm, *N* = 10 BAV‐ND and *N* = 11 TAV‐ND. Results are expressed as mean area positive staining (%) ± SEM (Students t‐test). *P < 0.05, **P < 0.01, ***P < 0.001, ****P ≤ 0.0001.

Because of its well‐known and established role in EndMT/EMT, as well as its high association with human cancers, we chose to focus our efforts on miR‐200c. First, *in situ* hybridization was conducted to identify the cellular source of miR‐200c expression. MiR‐200c expression was very low and could not be detected in the BAV aorta. A weak but discernible expression could however be detected in endothelial cells in dilated aorta of TAV patients, suggesting that endothelial cells are the major source of miR‐200c expression (Fig. 3a). Then, in order to investigate the co‐expression between different miR‐200 family members in the aortic endothelium, endothelial cells from BAV and TAV ascending aortas were isolated and miR‐200c, miR‐200b and miR‐429 expression levels were determined. Subsequent co‐expression analysis showed a high correlation between all miR‐200 family members (Table S5).

### Expression of the miR‐200 family is downregulated in BAV‐ND

To validate our *in silico* findings and gain further insight into the possible regulatory roles of the miR‐200 family in BAV aneurysm development, we performed expression analysis in both nondilated and dilated BAV and TAV aortas and compared expression levels of miR‐200 family members between the different patient groups, nondilated BAV (BAV‐ND) vs. nondilated TAV (TAV‐ND), and dilated BAV (BAV‐D) vs. dilated TAV (TAV‐D). The expression of miR‐200c, miR‐200b and miR‐429 was measured in the aortic intima–media portion using qRT‐PCR. This showed that only miR‐200c was significantly downregulated in BAV‐ND aorta compared to TAV‐ND aorta following adjustment for potential confounding factors, including age, valve disease and aortic dimensions (*P* = 0.037; Fig. 3b). MiR‐429 showed a tendency towards being downregulated in BAV‐ND following correction for confounding factors, although not reaching statistical significance (*P* = 0.113). There was no difference in miR‐200b expression between nondilate BAV and TAV aortas (data not shown). The expression of all three miRs was unchanged in dilated aortas between BAV and TAV (data not shown).

### Increased mRNA expression of the miR‐200 family targets ZEB1/2 in the BAV aorta

MiR‐200 family suppresses mRNA expression of the master EndMT/EMT transcription factors ZEB1 and ZEB2 via a negative feedback loop. This loop coordinates EndMT/EMT events through regulation of adherens junction protein, cadherin 30, 31. In order to confirm the inhibitory effect of miR‐200 family on the expression of key EMT transcription factors, we first used locked nucleic acid (LNA) to downregulate the expression of miR‐200 family in cultured endothelial cells (EA.hy926) and analysed the expression of SNAI1 and ZEB1. Figure S4 shows that both *SNAI1* and *ZEB1* expression was significantly upregulated upon miR‐200 family inhibition.

Then, the expression of ZEB1 and ZEB2 was analysed in the BAV and TAV ascending aorta. The mRNA expression of ZEB transcription factors showed an interesting difference between BAV and TAV patients. In accordance with the observed decreased expression of miR‐200 family in BAV patients, both ZEB1 and ZEB2 had significantly higher expression in nondilated aortas when compared to TAV‐ND (*P* = 0.0001 for ZEB1 and *P* = 0.0002 for ZEB2), but both transcription factors expression remained unchanged as a result of aneurysm in BAV‐D patients. ZEB1 expression, however, was significantly higher in BAV‐D when a comparison was made with TAV‐D aortas (*P* = 0.0003; Fig. 3c). These observations implied that (i) higher expression of ZEB1/2 in BAV is a preaneurysm event and (ii) in the absence of a substantial increase in TAV aneurysm, it remains still significantly higher in BAV‐D. In TAV, however, ZEB1 expression did not significantly shift in aneurysm (comparing TAV‐ND vs. TAV‐D), whilst ZEB2 expression increased as a result of aneurysm development (*P* = 0.0002; Fig. 3c), indicating (i) ZEB1 is not activated in TAV either before or after aneurysm development, (ii) ZEB2 activation is a postaneurysm event in TAV. We have previously shown significantly higher gene expression of ZEB1 in BAV and ZEB2 in TAV dilated patients by microarrays 3. Hence, we observed a key functional distinction between the two transcription factors with regard to the development of aneurysm in BAV and TAV patients. The increased expression of ZEB1 protein in BAV‐ND has previously been shown by IHC staining 5. Here, we further confirmed it by Western blot, showing a tendency towards higher ZEB1 expression in BAV‐ND (*P* = 0.07; Fig. S5). The elevated protein expression of ZEB2 in BAV‐ND was also confirmed by IHC using monoclonal anti‐ZEB2 antibody (*P* < 0.0001; Fig. 3d).

### Mir‐200c promoter has a higher occupancy by ZEB1/2 in BAV‐ND individuals

**Figure 4:**
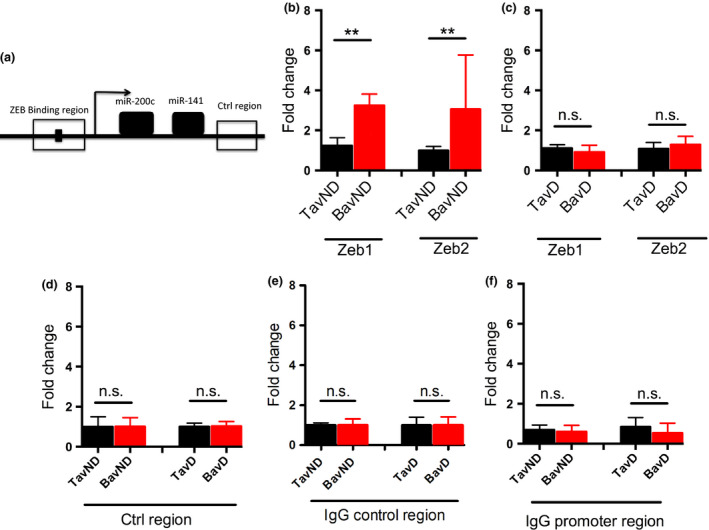
Chromatin Immunoprecipitation (ChIP) of ZEB occupancy on the miR‐200c/141 promoter region. (a) Schematic representation of the miR‐200c promoter, the region used for designing the ChIP primer sets (−480 to −210, and +872 to +995) and the known ZEB binding region, (b, c) qRT‐PCR of ChIP for ZEB1 and ZEB2, BAV and TAV nondilated (b) and dilated (c) aortic tissue samples, (d) control region, and (e, f) IgG‐positive control. *N* = 5 patients in each group. **P < 0.01 (Mann–Whitney *U*‐test).

The miR‐200c promoter contains several binding sites for transcription factors ZEB1/2 32. We used ChIP‐qRT‐PCR assay to make a comparison between the binding of these transcription factors to 200c promoter in nondilated aortas from BAV and TAV patients. Figure 4a shows a schematic representation of the miR‐200c promoter, including the known ZEB binding region. Our data revealed a significantly higher binding of ZEB1/2 to the promoter of miR‐200c in BAV‐ND compared to TAV‐ND (*P* = 0.008 for both ZEB1 and ZEB2; Fig. 4b), whilst binding of ZEB1/2 to control region did not differ between BAV and TAV nondilated aorta (Fig. 4d), confirming the specificity of binding to miR‐200c promoter. In dilated aortas, no binding difference was observed (Fig. 4c). IgG binding to both miR‐200c promoter region and the control region did not show any difference either in nondilated or in dilated BAV/TAV (Fig. 4e,f). We therefore concluded that ZEB/miR‐200 double‐negative feedback loop is activated in BAV‐ND and in this group of patients, the enhanced expression of ZEB1/2 is at least partly due to the suppression of miR‐200c by higher degree of ZEB1/2 binding to the miR‐200c promoter, that in turn increases the ZEB1/2 expression (Fig. 4g).

## Discussion

In this study, we used a network‐based approach to investigate the possible regulatory influence of miRNAs on pathogenic mechanisms that may underlie the higher susceptibility of BAV individuals to aneurysm development. MiR‐200 family was identified as the top‐ranked miRNAs, with downstream target genes spanning the entire network, and thus highly likely to act as a key regulator of aneurysm susceptibility in BAV individuals. The expression of miR‐200 family members was highly correlated, as shown in BAV and TAV patient aortic endothelial cells. We validated the *in silico* findings by qRT‐PCR in BAV and TAV patients having either a nondilated or dilated aorta. The significantly lower expression of miR‐200c, irrespective of potential confounding factors, in parallel with higher expression of the targets ZEB1/2, in nondilated BAV aorta compared to nondilated TAV, strongly suggests the induction of an EndMT/EMT‐like processes by a miR‐200c/ZEB negative feedback loop in the aortic wall of BAV patients prior to dilation. Interestingly, miR‐200c expression was only significantly decreased in BAV‐ND aortas, thus the retained higher expression of ZEB1 in BAV‐D aortas compared to TAV‐D aortas should be due to other regulatory mechanisms.

Furthermore, our results pinpointed several other interesting molecular features that differed between the development of aneurysm in BAV and TAV individuals. ZEB1 expression level was significantly higher in BAV‐D compared to TAV‐D aorta, whilst the expression levels were not different either between BAV‐ND and BAV‐D aorta or TAV‐ND and TAV‐D aorta, implying that ZEB1 activity did not increase due to the aneurysm development, either in BAV or in TAV patients. Thus, the high expression of ZEB1 in nondilated BAV aortas was most probably due to a pre‐existing condition. Moreover, ZEB2 showed a different expression pattern from ZEB1, that is being significantly higher in BAV‐ND compared to TAV‐ND whilst significantly higher in TAV‐D compared to BAV‐D, denoting a very sharp increased activity of ZEB2 in aneurysm development of TAV but not BAV. This is further supported by comparison of ZEB2 expression levels in TAV and BAV patients, dilated versus nondilated.

The function of ZEB1 and ZEB2 is largely overlapping regarding cancer‐related EMT 33. To our knowledge, this is the first observation reporting a functional difference between the two ZEB partners in the context of EndMT/EMT‐like processes. Higher ZEB1/ZEB2 expression, as well as a lower expression of the miR‐200 family, is compatible with an ongoing EndMT/EMT process in BAV that is aneurysm independent, and the transition to aneurysm state is most probably due to the further exasperation of a pre‐existing EndMT/EMT. In TAV, higher expression of ZEB2 may be due to fibrosis or induction of wound healing. In other word, the processes induced by ZEBs in BAV and TAV may be of different natures (for instance a class I EndMT/EMT in BAV and a class II in TAV) and hence could differ in the pattern of ZEB activation. In spite of functional overlap of ZEB1 and ZEB2, some operational differences between them have been reported. One such difference is their angiogenic properties. ZEB1 is the only major EMT transcription factor that has not been reported to be activated during angiogenesis 33 and has even been reported to be anti‐angiogenic 34. This interesting difference between BAV and TAV may reflect a particular role for ZEB1 activation at the onset of cardiac cushion EndMT (Type I) that may be important for the modulation of pro‐angiogenic VEGF signalling, the oscillation of which was shown to be critical for proper development of endocardial cushion and hence the aortic valves 35, 36. The distinction of ZEB function between BAV and TAV aneurysm may be hinting an interesting functional nonoverlap of ZEB proteins in different types of EndMT/EMT and deserves further experimental clarification. Moreover, ChIP analysis of ZEB1/2 binding to the promoter of mir‐200c established a higher occupancy of mir‐200c promoter by ZEB1/2 in BAV‐ND, but not in BAV‐D, further confirming the establishment of a negative feedback loop of mir‐200c/ZEBs only prior to dilation, and that its activation in BAV may be one of the initial steps of the EndMT/EMT process. The ChIP study, however, does not support a mir‐200c/ZEB loop being the regulatory mechanism behind the enhanced expression of ZEB2 in TAV‐D.

A literature search for the function of other highly ranked microRNAs (Table S4) indicated that, apart from the miR‐200 family already known to be crucial for EMT, all other top‐ranked miRNAs have been cited in prior studies relevant to EMT and/or cancer. Some of the miRNAs appearing on top of our nondilated and dilated list have been identified as ‘core regulatory microRNAs’ of EMT in various experimental setups. These major miRNAs include miR‐203, miR‐495, miR‐1, miR‐133, miR‐29, miR‐204, miR‐15/16, miR‐96, miR‐506, miR‐128, miR‐181 and miR‐27 37, 38, 39, 40, 41. However, in all these studies, the miR‐200 family appeared to be the strongest regulator of EMT. Additionally, miR‐145, miR‐181 and miR‐29 have been discovered in association with thoracic aortic aneurysm 7.

The appearance of EMT as the fifth and third hallmark in nondilated and dilated samples, respectively, implies that, although the miR‐200 family serves as a master regulator of EMT and governs such processes before dilation, the accomplishment of EMT in dilated samples needs cooperation of other miRNAs. For instance, cooperation of miR‐200c with miR‐203 is required for the suppression of stem cell factors in cancer cells and mouse embryonic stem (ES) cells 42, and miR‐203 establishes a regulatory loop with another transcription factor important for EMT 43. Hence, although the miR‐200 family is initially important, the collaboration of other EMT‐related microRNAs might be required for aneurysm development. Alternatively, initial expression of some microRNAs may facilitate the epigenetic modifications required for the promotion of EMT. Indeed, microRNAs have been shown to regulate, and be regulated by, the epigenetic machinery via DNA methylation and histone modifications 44. Mir‐200c directly targets DNMT3a and the two can mutually regulate each other in an epigenetic feedback loop by controlling DNA methylation 45. In support of this hypothesis, we have previously reported the increased expression of DNMT3a in nondilated BAV aorta 5. MiR‐200a also targets SIRT1 (a class III histone deacetylase) at the SIRT1 3′‐UTR forming a negative feedback loop to regulate histone acetylation and thereby EMT 46. Several other miRNAs included in our lists such as miR‐124, miR‐506 47 and miR‐29 48, 49 have also been shown to target the epigenetic machinery. This highlights the potential cooperation of microRNAs to reinforce each other and perform the biological processes that may be different, but will eventually arrive at the same endpoint.

## Conclusion

Here, we report a miR‐200‐dependent process of EndMT/EMT in ascending aortas of individuals with a BAV prior to aneurysm development. This process is initially supported by a miR‐200c/ZEB feedback loop and is most probably advanced by cooperation of other miRNAs. Considering the structural and molecular reorganization of cells during this process, induction of EndMT/EMT is a plausible biological mechanism rendering the BAV ascending aorta more prone to aneurysm development.

## Conflict of interest statement

S.Y. Chan has served as a consultant for Actelion (significant), Gilead, Pfizer and Vivus (modest). No competing financial interests are otherwise declared.

## Supporting information


**Table S1.** Patient characteristics
**Table S2.** Primer sequences, MiR200c promoter region and control region.
**Table S3.** MicroRNA spanning scores, BAV vs. TAV non‐dilated and dilated aorta, respectively.
**Table S4.** (A) Top 10 microRNAs according to spanning score, selected by *in silico* method. BAV vs. TAV non‐dilated aorta. (B) Top 10 microRNAs according to spanning score, selected by *in silico* method. BAV vs. TAV dilated aorta.
**Table S5.** Co‐expression of miR‐200b, miR‐200C and miR‐429 in BAV and TAV endothelial cells (Pearson correlation, R‐values).
**Figure S1.** Electron microscopy view of autophogosome‐like structure.
**Figure S2.** Computational network analysis of dilated BAV and TAV aorta.
**Figure S3.** Hallmark analysis of dilated network genes, BAV vs. TAV.
**Figure S4.** ZEB1 and Snai1 expression in cultured endothelial cells (EA.hy926) after miR‐200 **family inhibition.**
**Figure S5.** ZEB1 expression in BAV and TAV non‐dilated aortas.
