# Supplementary material for: The mir‐200 family regulates key pathogenic events in ascending aortas of individuals with bicuspid aortic valves
Source: J Intern Med. 2018 Oct 2;285(1):102–14. doi: 10.1111/joim.12833 (PMC6488227; doi:10.1111/joim.12833)
Supplement: Supplementary file 1 — Table S1. Patient characteristics Table S2. Primer sequences, MiR200c promoter region and control region. Table S3. MicroRNA spanning scores, BAV vs. TAV non‐dilated and dilated aorta, respectively. Table S4. (A) Top 10 microRNAs according to spanning score, selected by in silico method. BAV vs. TAV non‐dilated aorta. (B) Top 10 microRNAs according to spanning score, selected by in silico method. BAV vs. TAV dilated aorta. Table S5. Co‐expression of miR‐200b, miR‐200C and miR‐429 in BAV and TAV endothelial cells (Pearson correlation, R‐values). Figure S1. Electron microscopy view of autophogosome‐like structure. Figure S2. Computational network analysis of dilated BAV and TAV aorta. Figure S3. Hallmark analysis of dilated network genes, BAV vs. TAV. Figure S4. ZEB1 and Snai1 expression in cultured endothelial cells (EA.hy926) after miR‐200 family inhibition. Figure S5. ZEB1 expression in BAV and TAV non‐dilated aortas. [file JOIM-285-102-s001.pdf]

# Supplementary Materials

## The mir-200 family regulates key pathogenic events in ascending aortas of individuals with bicuspid aortic valves

Short title: Mir-200 family in patients with BAV

Shohreh Maleki[1], Katherine A. Cottrill[2], Flore-Anne Poujade[1], Anirban Bhattachariya[1], Otto Bergman[1], Jesper R. Gådin[1], Nancy Simon[1], Karin Lundströmer[1], Anders Franco-Cereceda[3], Hanna M. Björck[1], Stephen Y. Chan[2], Per Eriksson[1]

[1]Cardiovascular Medicine Unit, Center for Molecular Medicine, Department of Medicine, Karolinska Institutet, Karolinska University Hospital Solna, Stockholm, Sweden [2]Pittsburgh Heart, Lung, Blood, and Vascular Medicine Institute, Division of Cardiology, Department of Medicine, University of Pittsburgh School of Medicine and University of Pittsburgh Medical Center, Pittsburgh, PA USA [3]Cardiothoracic Surgery Unit, Department of Molecular Medicine and Surgery, Karolinska Institutet, Stockholm, Sweden

| **Page** | **Content** |
|---|---|
| 2 | Supplementary Table S1, Patient characteristics |
| 2 | Supplementary Table S2, Primer sequences, MiR200c promoter region and control region |
| 2 | Supplementary Table S3, MicroRNA spanning scores, BAV vs. TAV non-dilated and dilated aorta, respectively |
| 3 | Supplementary Table S4A, Top 10 microRNAs according to spanning score, BAV vs. TAV non-dilated aorta |
| 3 | Supplementary Table S4B, Top 10 microRNAs according to spanning score, BAV vs. TAV dilated aorta |
| 3 | Supplementary Table S5. Co-expression analysis miR-200b, miR-200C and miR-429 in BAV and TAV endothelial cells. |
| 4 | Supplementary Fig. S1, Electron microscopy autophogosome-like structure, BAV non-dilated aorta |
| 5 | Supplementary Fig. S2, Computational network analysis, dilated aorta |
| 6 | Supplementary Fig. S3, Enrichment analysis of hallmark gene sets, dilated network |
| 7 | Supplementary Fig. S4, LNA mir200-zeb-snai |
| 8 | Supplementary Fig. S5, Western blot, ZEB1 expression, BAV and TAV non-dilated aorta |
| 9 | References |

**Supplementary Table S1.** Patient characteristics

| Patient characteristics | | | | | | |
|---|---|---|---|---|---|---|
| | **BAV-ND** | **TAV-ND** | ***P*** | **BAV-D** | **TAV-D** | ***P*** |
| **N** | 45 | 45 | | 21 | 18 | |
| **Age, years** | 60 (13) | 68 (12) | <0.01 | 58 (13) | 68 (5) | <0.01 |
| **Male gender** | 33 | 26 | 0.12 | 16 | 11 | 0.49 |
| **BSA, $m^2$** | 1.98 (0.18) | 1.95 (0.21) | 0.36 | 2.06 (0.24) | 2.04 (0.25) | 0.80 |
| **Triglycerides, mmol/L** | 1.23 (0.60) | 1.11 (0.37) | 0.25 | 1.69 (1.13) | 1.02 (0.37) | 0.03 |
| **Cholesterol, mmol/L** | 4.45 (1.01) | 4.84 (1.20) | 0.10 | 5.25 (0.97) | 5.14 (0.91) | 0.74 |
| **hsCRP, mg/L** | 2.34 (3.23) | 2.87 (3.83) | 0.48 | 2.45 (3.30) | 3.22 (4.11) | 0.53 |
| **Systolic BP, mmHg** | 131 (15) | 138 (22) | 0.10 | 132 (18) | 145 (18) | 0.03 |
| **Diastolic BP, mmHg** | 78 (11) | 77 (15) | 0.91 | 82 (10) | 74 (11) | 0.03 |
| **Diabetes** | 5 | 4 | 0.74 | 1 | 0 | 1.00 |
| **Aortic aneurysm and valve disease** | | | | | | |
| **Ascending aortic aneurysm, mm** | 32.9 (4.9) | 31.2 (4.1) | 0.08 | 49.6 (5.1) | 53.6 (4.6) | 0.04 |
| **Aortic valve stenosis** | 39 | 28 | <0.05 | 12 | 0 | <0.001 |
| **Aortic valve regurgitation** | 5 | 8 | 0.55 | 7 | 13 | <0.05 |

Non-dilated (ND) ascending aorta <40mm; Dilated (D) ascending aorta >45mm. BSA, body surface area; hsCRP, high-sensitive C-reactive protein; BP, blood pressure. Diabetes was recorded via questionnaire of medical history. Continuous variables are presented as mean (SD) and ordinal variables are presented as n. *P* indicates significance for differences between groups using Student's t-test for continuous variables and a Fisher's exact test for nominal variables.

**Supplementary Table S2.** Primer sequences, MiR200c promoter region and control region.

| **Oligo Name** | **Sequence (written 5' - 3')** | **Length** |
|---|---|---|
| Forward (200c promoter) | CTTAAAGCCCCTTCGTCTCC | 20 |
| Reverse (200c promoter) | GATTTACCCACCCTCATCCC | 20 |
| Forward (200c ctrl) | GCTCAGTGCTTCTCCAAGGT | 20 |
| Reverse (200c ctrl) | TAAGGACAGGGCCAGGGATT | 20 |

**Supplementary Table S3.** MicroRNA spanning scores, BAV vs. TAV non-dilated and dilated aorta, respectively.

*See separate pdf-file*

**Supplementary Table S4A.** Top 10 microRNAs according to spanning score, selected by *in silico* method. BAV vs. TAV non-dilated aorta.

| miRNA | Score | Genes targeted | Clusters targeted | P-value | References |
|---|---|---|---|---|---|
| miR-200bc/429/548a | 2,875 | 44 | 7 | 0,0001 | *See manuscript text* |
| miR-27abc/27a-3p | 2,471213 | 41 | 7 | 0,0022 | [1, 2] |
| miR-135ab/135a-5p | 2,264296 | 29 | 7 | 0,0012 | [3, 4] |
| miR-495/1192 | 2,256448 | 31 | 8 | 0,0062 | [5, 6] |
| miR-204/204b/211 | 2,251015 | 28 | 7 | 0,0011 | [7-11] |
| miR-133abc | 2,210151 | 27 | 7 | 0,0013 | [12] |
| miR-300/381/539-3p | 2,171962 | 31 | 8 | 0,0135 | [13-17] |
| miR-128/128ab | 2,170606 | 34 | 7 | 0,0081 | [18-20] |
| miR-124/124ab/506 | 2,167087 | 42 | 8 | 0,1412 | [12, 21-24] [25, 26] |
| miR-29abcd | 2,099049 | 33 | 7 | 0,0127 | [27-29] |

**Supplementary Table S4B.** Top 10 microRNAs according to spanning score, selected by *in silico* method. BAV vs. TAV dilated aorta.

| miRNA | Score | Genes targeted | Clusters targeted | P-value | References |
|---|---|---|---|---|---|
| miR-124/124ab/506 | 2,924743 | 152 | 8 | 0,0002 | [30, 31] |
| miR-23abc/23b-3p | 2,75 | 114 | 8 | <0.0001 | [32] |
| miR-200bc/429/548a | 2,480263 | 111 | 6 | <0.0001 | *See manuscript text* |
| miR-1ab/206/613 | 2,434211 | 85 | 7 | <0.0001 | [33-38] |
| miR-181abcd/4262 | 2,275457 | 117 | 5 | 0,0003 | [39, 40] |
| miR-144 | 2,236842 | 93 | 5 | <0.0001 | [41, 42] |
| miR-203 | 2,20234 | 81 | 8 | 0,0021 | [43-46] |
| miR-15abc/16/16abc/195/322/424/497/1907 | 2,166147 | 115 | 6 | 0,0023 | [47-52] |
| miR-145 | 2,144737 | 79 | 5 | 0,0001 | [53-55] |
| miR-96/507/1271 | 2,12781 | 97 | 6 | 0,0011 | [56-59] |

**Supplementary Table S5.** Co-expression of miR-200b, miR-200C and miR-429 in BAV and TAV endothelial cells (Pearson correlation, R-values).

| | **miR-200b** | **miR-429** | **miR-200c** |
|---|---|---|---|
| **miR-200b** | 1 | 0,499 | 0,433 |
| **miR-429** | 0,499 | 1 | 0,569 |
| **miR-200c** | 0,433 | 0,569 | 1 |

## Supplementary Figure S1

***Electron microscopy view of autophogosome-like structure.*** BAV endothelial cell membrane, non-dilated aorta. EC: Endothelial cells; SMC: Smooth muscle cells, (B) is the higher magnification of (A).

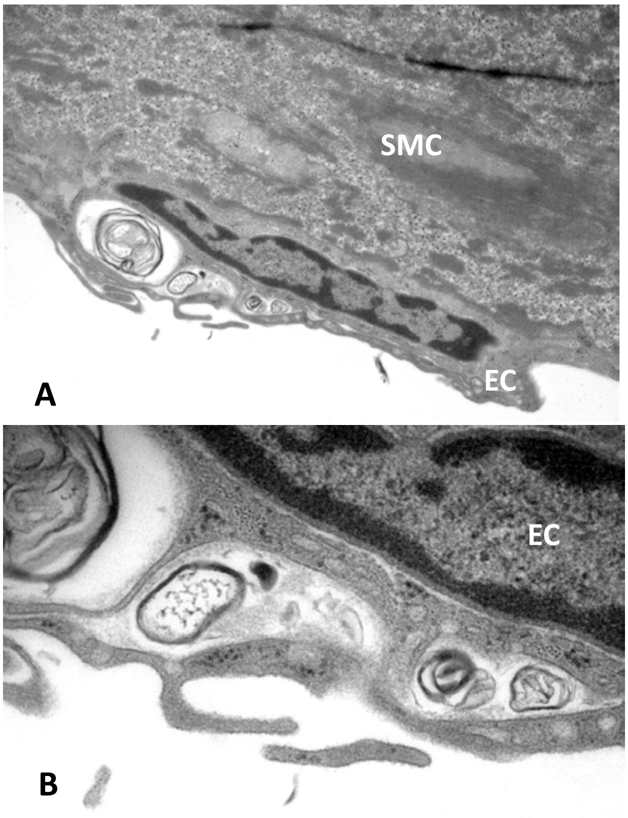

## Supplementary Figure S2

***Computational network analysis of dilated BAV and TAV aorta.*** Cluster modules of highly interacting groups of genes in the dilated (D) network. Different colors in the network represent different cluster modules (non-labeled). The network was clustered using a spectral partition-based algorithm, as implemented in the ReactomeFI tool for Cytoscape. miRNA targets were cross-referenced to the network and regulatory miRNAs were identified and ranked according to calculated spanning scores (see materials and methods for details). Node size represents spanning scores of each gene. Module assignments/labels and spanning sores are provided in supplementary Table S2B and S3.

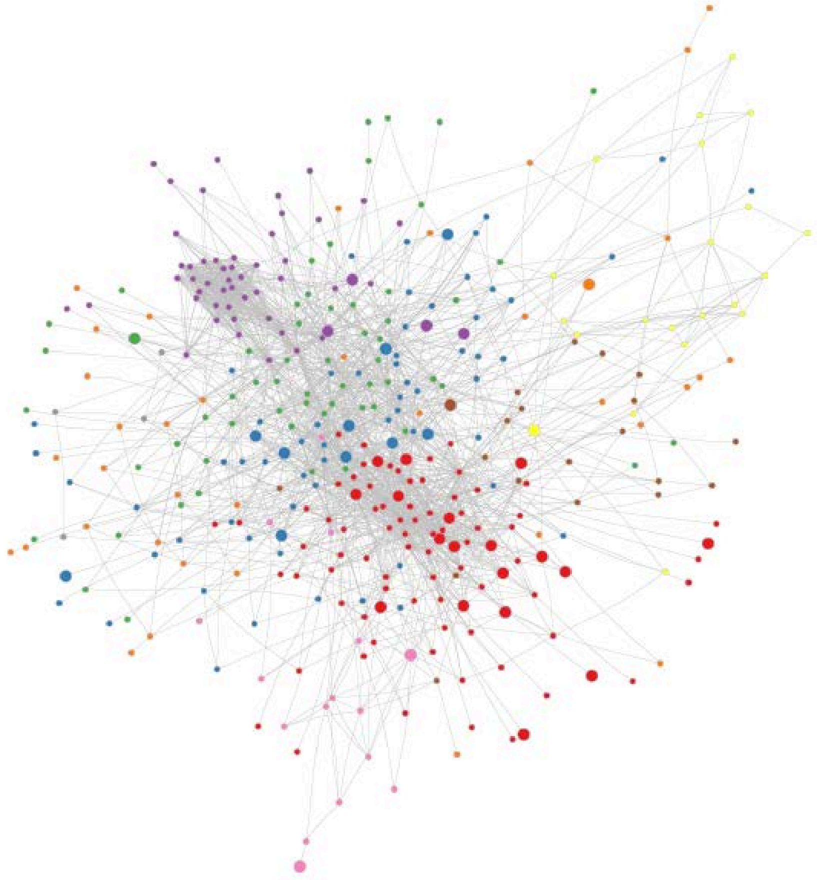

## Supplementary Figure S3

***Hallmark analysis of dilated network genes, BAV vs. TAV.*** -logP indicates the enrichment p-value (hypergeometric test). Red dashed line indicates p-value 0.05; green dashed line indicates FDR-adjusted p-value 0.05. Hallmark gene sets are curated by the Molecular Signatures Database (MSigDB).

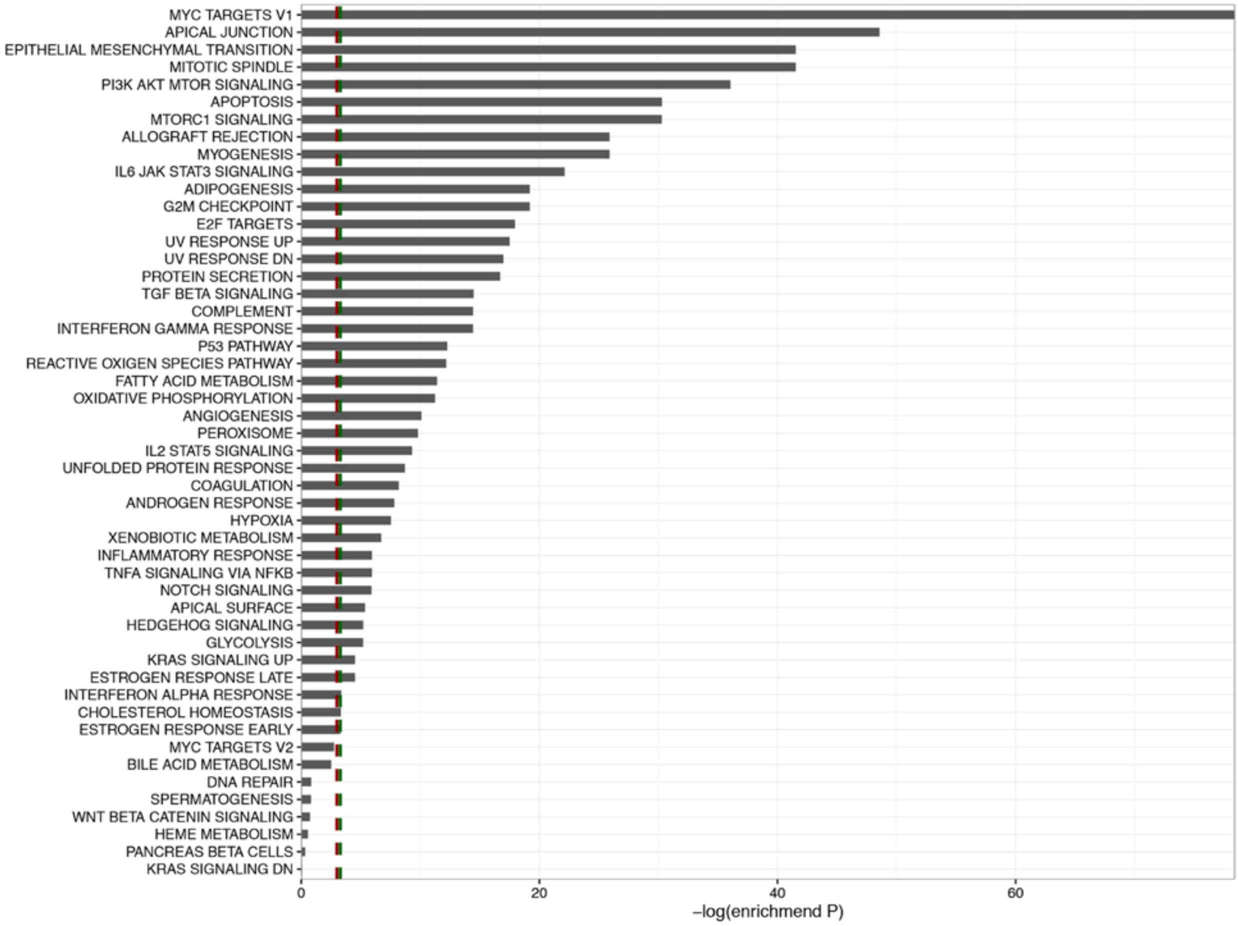

## Supplementary Figure S4

***ZEB1 and Snai1 expression in cultured endothelial cells (EA.hy926) after miR-200 family inhibition.***
Locked nucleic acid (LNA) were used for miR200 family down-regulation**.** Data are presented as fold change of control ± SEM (Student's t-test).

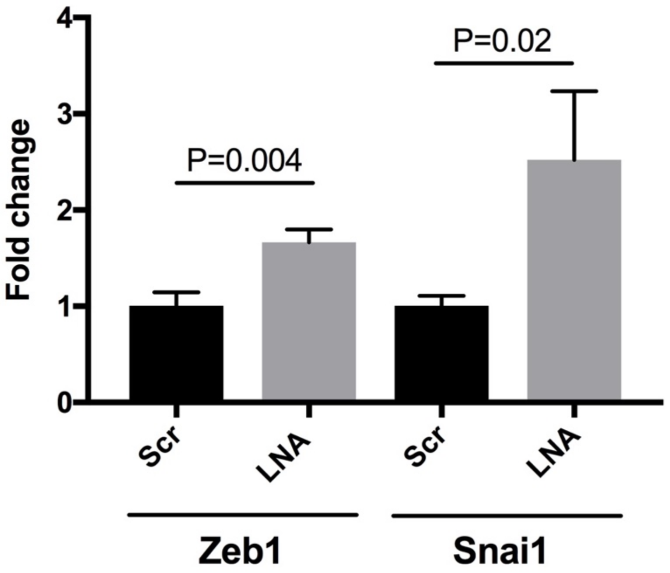

## Supplementary Figure S5

***ZEB1 expression in BAV and TAV non-dilated aortas.*** Western blot analysis of 20 µg protein extract using anti-ZEB1 antibody***.*** Data are shown as mean ZEB1/Actinβ ratio ± SEM. N=15 BAV and N=16 TAV (Student's t-test).

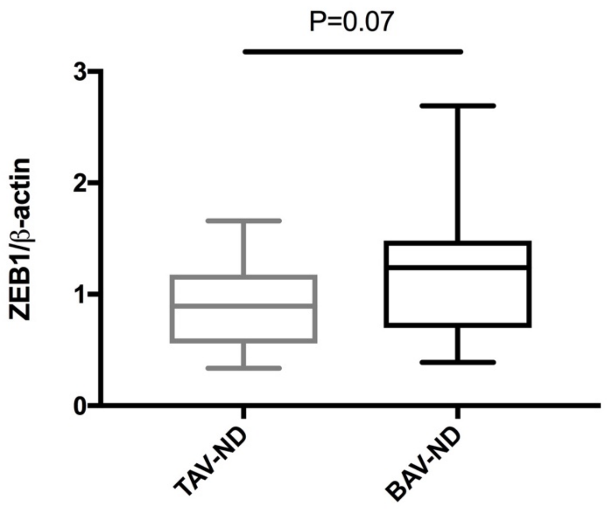

## References


1. Suzuki HI, Katsura A, Mihira H, Horie M, Saito A, Miyazono K. Regulation of TGF-beta-mediated endothelial-mesenchymal transition by microRNA-27. *J Biochem* 2017; **161**: 417-20.

2. Zhang Z, Liu S, Shi R, Zhao G. miR-27 promotes human gastric cancer cell metastasis by inducing epithelial-to-mesenchymal transition. *Cancer Genet* 2011; **204**: 486-91.

3. Golubovskaya VM, Sumbler B, Ho B, Yemma M, Cance WG. MiR-138 and MiR-135 directly target focal adhesion kinase, inhibit cell invasion, and increase sensitivity to chemotherapy in cancer cells. *Anticancer Agents Med Chem* 2014; **14**: 18-28.

4. Taipaleenmaki H, Browne G, Akech J *et al.* Targeting of Runx2 by miR-135 and miR-203 Impairs Progression of Breast Cancer and Metastatic Bone Disease. *Cancer Res* 2015; **75**: 1433-44.

5. Wei T, Zhu W, Fang S *et al.* miR-495 promotes the chemoresistance of SCLC through the epithelial-mesenchymal transition via Etk/BMX. *Am J Cancer Res* 2017; **7**: 628-46.

6. Haga CL, Phinney DG. MicroRNAs in the imprinted DLK1-DIO3 region repress the epithelial-to-mesenchymal transition by targeting the TWIST1 protein signaling network. *J Biol Chem* 2012; **287**: 42695-707.

7. Wang Y, Zhou Y, Yang Z *et al.* MiR-204/ZEB2 axis functions as key mediator for MALAT1-induced epithelial-mesenchymal transition in breast cancer. *Tumour Biol* 2017; **39**: 1010428317690998.

8. Chen SJ, Wu P, Sun LJ *et al.* miR-204 regulates epithelial-mesenchymal transition by targeting SP1 in the tubular epithelial cells after acute kidney injury induced by ischemia-reperfusion. *Oncol Rep* 2017; **37**: 1148-58.

9. Li W, Miao X, Liu L *et al.* Methylation-mediated silencing of microRNA-211 promotes cell growth and epithelial to mesenchymal transition through activation of the AKT/beta-catenin pathway in GBM. *Oncotarget* 2017; **8**: 25167-76.

10. Xu D, Liu S, Zhang L, Song L. MiR-211 inhibits invasion and epithelial-to-mesenchymal transition (EMT) of cervical cancer cells via targeting MUC4. *Biochem Biophys Res Commun* 2017; **485**: 556-62.

11. Yu H, Yang W. MiR-211 is epigenetically regulated by DNMT1 mediated methylation and inhibits EMT of melanoma cells by targeting RAB22A. *Biochem Biophys Res Commun* 2016; **476**: 400-05.

12. Xiao B, Liu H, Gu Z, Ji C. Expression of microRNA-133 inhibits epithelial-mesenchymal transition in lung cancer cells by directly targeting FOXQ1. *Arch Bronconeumol* 2016; **52**: 505-11.

13. Yu J, Xie F, Bao X, Chen W, Xu Q. miR-300 inhibits epithelial to mesenchymal transition and metastasis by targeting Twist in human epithelial cancer. *Mol Cancer* 2014; **13**: 121.

14. Wu MH, Huang PH, Hsieh M, Tsai CH, Chen HT, Tang CH. Endothelin-1 promotes epithelial-mesenchymal transition in human chondrosarcoma cells by repressing miR-300. *Oncotarget* 2016; **7**: 70232-46.

15. Xue Y, Xu W, Zhao W, Wang W, Zhang D, Wu P. miR-381 inhibited breast cancer cells proliferation, epithelial-to-mesenchymal transition and metastasis by targeting CXCR4. *Biomed Pharmacother* 2017; **86**: 426-33.

16. He X, Wei Y, Wang Y, Liu L, Wang W, Li N. MiR-381 functions as a tumor suppressor in colorectal cancer by targeting Twist1. *Onco Targets Ther* 2016; **9**: 1231-9.

17. Hu WW, Chen PC, Chen JM *et al.* Periostin promotes epithelial-mesenchymal transition via the MAPK/miR-381 axis in lung cancer. *Oncotarget* 2017; **8**: 62248-60.

18. Yu WW, Jiang H, Zhang CT, Peng Y. The SNAIL/miR-128 axis regulated growth, invasion, metastasis, and epithelial-to-mesenchymal transition of gastric cancer. *Oncotarget* 2017; **8**: 39280-95.

19. Liu X, Liang Z, Gao K *et al.* MicroRNA-128 inhibits EMT of human osteosarcoma cells by directly targeting integrin alpha2. *Tumour Biol* 2016; **37**: 7951-7.

20. Fu J, Rodova M, Nanta R *et al.* NPV-LDE-225 (Erismodegib) inhibits epithelial mesenchymal transition and self-renewal of glioblastoma initiating cells by regulating miR-21, miR-128, and miR-200. *Neuro Oncol* 2013; **15**: 691-706.

21. Li SL, Gao HL, Lv XK *et al.* MicroRNA-124 inhibits cell invasion and epithelial-mesenchymal transition by directly repressing Snail2 in gastric cancer. *Eur Rev Med Pharmacol Sci* 2017; **21**: 3389-96.

22. Zhang X, Cai D, Meng L, Wang B. MicroRNA-124 inhibits proliferation, invasion, migration and epithelial-mesenchymal transition of cervical carcinoma cells by targeting astrocyte-elevated gene-1. *Oncol Rep* 2016; **36**: 2321-8.

23. Ma T, Zhao Y, Wei K *et al.* MicroRNA-124 Functions as a Tumor Suppressor by Regulating CDH2 and Epithelial-Mesenchymal Transition in Non-Small Cell Lung Cancer. *Cell Physiol Biochem* 2016; **38**: 1563-74.

24. Li Z, Liu Z, Dong S *et al.* miR-506 Inhibits Epithelial-to-Mesenchymal Transition and Angiogenesis in Gastric Cancer. *Am J Pathol* 2015; **185**: 2412-20.

25. Sakimura S, Sugimachi K, Kurashige J *et al.* The miR-506-Induced Epithelial-Mesenchymal Transition is Involved in Poor Prognosis for Patients with Gastric Cancer. *Ann Surg Oncol* 2015; **22 Suppl 3**: S1436-43.

26. Sun Y, Mezzanzanica D, Zhang W. MiR-506: A Multitasker in Suppression of the Epithelial-to-Mesenchymal Transition. *RNA Dis* 2014; **1**: e447.

27. Cicchini C, de Nonno V, Battistelli C *et al.* Epigenetic control of EMT/MET dynamics: HNF4alpha impacts DNMT3s through miRs-29. *Biochim Biophys Acta* 2015; **1849**: 919-29.

28. Rostas JW, 3rd, Pruitt HC, Metge BJ *et al.* microRNA-29 negatively regulates EMT regulator N-myc interactor in breast cancer. *Mol Cancer* 2014; **13**: 200.

29. Li M, Li H, Liu X, Xu D, Wang F. MicroRNA-29b regulates TGF-beta1-mediated epithelial-mesenchymal transition of retinal pigment epithelial cells by targeting AKT2. *Exp Cell Res* 2016; **345**: 115-24.

30. Qin W, Pan Y, Zheng X *et al.* MicroRNA-124 regulates TGF-alpha-induced epithelial-mesenchymal transition in human prostate cancer cells. *Int J Oncol* 2014; **45**: 1225-31.

31. Li Z, Wang X, Li W, Wu L, Chang L, Chen H. miRNA-124 modulates lung carcinoma cell migration and invasion. *Int J Clin Pharmacol Ther* 2016; **54**: 603-12.

32. Yang L, Fan Y, Zhang X, Ma J. miRNA-23 regulates high glucose induced epithelial to mesenchymal transition in human mesotheial peritoneal cells by targeting VDR. *Exp Cell Res* 2017; **360**: 375-83.

33. Xu L, Zhang Y, Wang H, Zhang G, Ding Y, Zhao L. Tumor suppressor miR-1 restrains epithelial-mesenchymal transition and metastasis of colorectal carcinoma via the MAPK and PI3K/AKT pathway. *J Transl Med* 2014; **12**: 244.

34. Liu YN, Yin JJ, Abou-Kheir W *et al.* MiR-1 and miR-200 inhibit EMT via Slug-dependent and tumorigenesis via Slug-independent mechanisms. *Oncogene* 2013; **32**: 296-306.

35. Yin K, Yin W, Wang Y *et al.* MiR-206 suppresses epithelial mesenchymal transition by targeting TGF-beta signaling in estrogen receptor positive breast cancer cells. *Oncotarget* 2016; **7**: 24537-48.

36. Chen QY, Jiao DM, Wang J *et al.* miR-206 regulates cisplatin resistance and EMT in human lung adenocarcinoma cells partly by targeting MET. *Oncotarget* 2016; **7**: 24510-26.

37. Chen QY, Jiao DM, Wu YQ *et al.* MiR-206 inhibits HGF-induced epithelial-mesenchymal transition and angiogenesis in non-small cell lung cancer via c-Met /PI3k/Akt/mTOR pathway. *Oncotarget* 2016; **7**: 18247-61.

38. Lima CR, Gomes CC, Santos MF. Role of microRNAs in endocrine cancer metastasis. *Mol Cell Endocrinol* 2017; **456**: 62-75.

39. Wang H, Tao T, Yan W *et al.* Upregulation of miR-181s reverses mesenchymal transition by targeting KPNA4 in glioblastoma. *Sci Rep* 2015; **5**: 13072.

40. Cai ZG, Zhang SM, Zhang H, Zhou YY, Wu HB, Xu XP. Aberrant expression of microRNAs involved in epithelial-mesenchymal transition of HT-29 cell line. *Cell Biol Int* 2013; **37**: 669-74.

41. Pan Y, Zhang J, Fu H, Shen L. miR-144 functions as a tumor suppressor in breast cancer through inhibiting ZEB1/2-mediated epithelial mesenchymal transition process. *Onco Targets Ther* 2016; **9**: 6247-55.

42. Li B, Zhang S, Shen H, Li C. MicroRNA-144-3p suppresses gastric cancer progression by inhibiting epithelial-to-mesenchymal transition through targeting PBX3. *Biochem Biophys Res Commun* 2017; **484**: 241-47.

43. Qu Y, Li WC, Hellem MR *et al.* MiR-182 and miR-203 induce mesenchymal to epithelial transition and self-sufficiency of growth signals via repressing SNAI2 in prostate cells. *Int J Cancer* 2013; **133**: 544-55.

44. Hu H, Xu Z, Li C *et al.* MiR-145 and miR-203 represses TGF-beta-induced epithelial-mesenchymal transition and invasion by inhibiting SMAD3 in non-small cell lung cancer cells. *Lung Cancer* 2016; **97**: 87-94.

45. Duan X, Fu Z, Gao L *et al.* Direct interaction between miR-203 and ZEB2 suppresses epithelial-mesenchymal transition signaling and reduces lung adenocarcinoma chemoresistance. *Acta Biochim Biophys Sin (Shanghai)* 2016; **48**: 1042-49.

46. Zhao G, Guo Y, Chen Z *et al.* miR-203 Functions as a Tumor Suppressor by Inhibiting Epithelial to Mesenchymal Transition in Ovarian Cancer. *J Cancer Sci Ther* 2015; **7**: 34-43.

47. Shi L, Jackstadt R, Siemens H, Li H, Kirchner T, Hermeking H. p53-induced miR-15a/16-1 and AP4 form a double-negative feedback loop to regulate epithelial-mesenchymal transition and metastasis in colorectal cancer. *Cancer Res* 2014; **74**: 532-42.

48. Takagi K, Yamakuchi M, Matsuyama T *et al.* IL-13 enhances mesenchymal transition of pulmonary artery endothelial cells via down-regulation of miR-424/503 in vitro. *Cell Signal* 2017; **42**: 270-80.

49. Mizrahi A, Barzilai A, Gur-Wahnon D *et al.* Alterations of microRNAs throughout the malignant evolution of cutaneous squamous cell carcinoma: the role of miR-497 in epithelial to mesenchymal transition of keratinocytes. *Oncogene* 2017;

50. Zhang N, Shen Q, Zhang P. miR-497 suppresses epithelial-mesenchymal transition and metastasis in colorectal cancer cells by targeting fos-related antigen-1. *Onco Targets Ther* 2016; **9**: 6597-604.

51. Wu X, Ruan Y, Jiang H, Xu C. MicroRNA-424 inhibits cell migration, invasion, and epithelial mesenchymal transition by downregulating doublecortin-like kinase 1 in ovarian clear cell carcinoma. *Int J Biochem Cell Biol* 2017; **85**: 66-74.

52. Liu C, Guan H, Wang Y *et al.* miR-195 Inhibits EMT by Targeting FGF2 in Prostate Cancer Cells. *PLoS One* 2015; **10**: e0144073.

53. Mei LL, Wang WJ, Qiu YT, Xie XF, Bai J, Shi ZZ. miR-145-5p Suppresses Tumor Cell Migration, Invasion and Epithelial to Mesenchymal Transition by Regulating the Sp1/NF-kappaB Signaling Pathway in Esophageal Squamous Cell Carcinoma. *Int J Mol Sci* 2017; **18**:

54. Li C, Lu L, Feng B *et al.* The lincRNA-ROR/miR-145 axis promotes invasion and metastasis in hepatocellular carcinoma via induction of epithelial-mesenchymal transition by targeting ZEB2. *Sci Rep* 2017; **7**: 4637.

55. Xiang Y, Zhang Y, Tang Y, Li Q. MALAT1 Modulates TGF-beta1-Induced Endothelial-to-Mesenchymal Transition through Downregulation of miR-145. *Cell Physiol Biochem* 2017; **42**: 357-72.

56. Zhang W, Qian P, Zhang X *et al.* Autocrine/Paracrine Human Growth Hormone-stimulated MicroRNA 96-182-183 Cluster Promotes Epithelial-Mesenchymal Transition and Invasion in Breast Cancer. *J Biol Chem* 2015; **290**: 13812-29.

57. Li XL, Hara T, Choi Y *et al.* A p21-ZEB1 complex inhibits epithelial-mesenchymal transition through the microRNA 183-96-182 cluster. *Mol Cell Biol* 2014; **34**: 533-50.

58. Liu H, Wang H, Liu X, Yu T. miR-1271 inhibits migration, invasion and epithelial-mesenchymal transition by targeting ZEB1 and TWIST1 in pancreatic cancer cells. *Biochem Biophys Res Commun* 2016; **472**: 346-52.

59. Xiang XJ, Deng J, Liu YW *et al.* MiR-1271 Inhibits Cell Proliferation, Invasion and EMT in Gastric Cancer by Targeting FOXQ1. *Cell Physiol Biochem* 2015; **36**: 1382-94.
