# Supplementary material for: The mir‐200 family regulates key pathogenic events in ascending aortas of individuals with bicuspid aortic valves
Source: J Intern Med. 2018 Oct 2;285(1):102–14. doi: 10.1111/joim.12833 (PMC6488227; doi:10.1111/joim.12833)
Supplement: Supplementary file 2 [file JOIM-285-102-s002.pdf]

Supplementary Table S3 - BAV vs. TAV non-dilated aorta				
**miRNA**	**Score**	**Genes Targeted**	**Clusters Targeted**	**pval**
miR-200bc/429/548a	2,875	44	7	0,0001
miR-27abc/27a-3p	2,471213	41	7	0,0022
miR-135ab/135a-5p	2,264296	29	7	0,0012
miR-495/1192	2,256448	31	8	0,0062
miR-204/204b/211	2,251015	28	7	0,0011
miR-133abc	2,210151	27	7	0,0013
miR-300/381/539-3p	2,171962	31	8	0,0135
miR-128/128ab	2,170606	34	7	0,0081
miR-124/124ab/506	2,167087	42	8	0,1412
miR-29abcd	2,099049	33	7	0,0127
miR-181abcd/4262	2,072205	34	8	0,0634
miR-144	2,066276	31	7	0,0113
miR-203	2,041764	28	8	0,0239
miR-130ac/301ab/301b/301b-3p/454/721/4295/3666	2,030016	30	7	0,0128
miR-590-3p	2,00667	35	7	0,0452
miR-96/507/1271	1,979934	30	8	0,0642
miR-30abcdef/30abe-5p/384-5p	1,968356	34	8	0,165
miR-182	1,956628	31	8	0,0981
miR-218/218a	1,92787	29	7	0,0266
miR-101/101ab	1,896797	27	7	0,0233
miR-17/17-5p/20ab/20b-5p/93/106ab/427/518a-3p/519d	1,890306	31	8	0,1807
miR-15abc/16/16abc/195/322/424/497/1907	1,88889	32	8	0,2257
miR-9/9ab	1,799626	32	7	0,1624
miR-145	1,768518	25	6	0,0158
miR-216a	1,768439	14	7	0,005
miR-125a-5p/125b-5p/351/670/4319	1,766196	26	6	0,0199
miR-19ab	1,760178	28	8	0,3197
miR-23abc/23b-3p	1,746462	32	6	0,0838
miR-148ab-3p/152	1,720341	26	5	0,0096
miR-24/24ab/24-3p	1,705977	22	6	0,015
miR-539/539-5p	1,654508	19	8	0,1286
miR-1ab/206/613	1,641716	25	6	0,0508
miR-25/32/92abc/363/363-3p/367	1,622452	24	7	0,1556
miR-340-5p	1,547772	28	7	0,7151
miR-873	1,495062	12	7	0,0408
miR-155	1,482918	15	7	0,0855
miR-26ab/1297/4465	1,476837	21	7	0,3175
miR-142-3p	1,449795	15	5	0,0116
miR-137/137ab	1,395367	25	6	0,4912
miR-205/205ab	1,385283	17	4	0,0101
miR-149	1,372434	15	6	0,0748
let-7/98/4458/4500	1,365062	20	7	0,721
miR-488	1,336215	13	6	0,0687

miR-150/5127	1,333605	11	6	0,0463
miR-290-5p/292-5p/371-5p/293	1,323931	12	6	0,0624
miR-153	1,292671	18	6	0,2922
miR-183	1,29151	13	6	0,1037
miR-494	1,288379	16	6	0,2
miR-374ab	1,277087	19	5	0,1315
miR-7/7ab	1,275943	12	7	0,307
miR-410/344de/344b-1-3p	1,267152	19	5	0,1441
miR-377	1,259961	17	5	0,1013
miR-433	1,256027	11	6	0,0946
miR-186	1,241943	15	7	0,7868
miR-455-5p	1,234776	8	6	0,0614
miR-202-3p	1,220674	17	6	0,46
miR-326/330/330-5p	1,218501	14	5	0,0792
miR-103a/107/107ab	1,207463	16	6	0,4214
miR-214/761/3619-5p	1,141398	17	5	0,3019
miR-132/212/212-3p	1,13755	13	5	0,1354
miR-129-5p/129ab-5p	1,133316	13	6	0,4452
miR-22/22-3p	1,124115	16	4	0,0908
miR-31	1,119835	10	6	0,269
miR-329/329ab/362-3p	1,115886	12	4	0,0424
miR-190/190ab	1,114105	8	5	0,059
miR-93/93a/105/106a/291a-3p/294/295/302abcde/372/373/428/519a/520be/520acd-3p/1378/1420ac	1,111441	15	6	0,8277
miR-223	1,103081	9	6	0,2546
miR-320abcd/4429	1,10274	20	4	0,2554
miR-194	1,095269	10	6	0,3373
miR-342-3p	1,087666	11	4	0,0446
miR-425/425-5p/489	1,079697	8	5	0,081
miR-33a-3p/365/365-3p	1,062156	10	5	0,1447
miR-140/140-5p/876-3p/1244	1,057493	12	4	0,0726
miR-335/335-5p	1,051235	10	4	0,0506
miR-138/138ab	1,047543	11	6	0,6454
miR-592/599	1,046431	9	4	0,0429
miR-141/200a	1,021289	18	4	0,3558
miR-543	1,008182	15	5	0,6775
miR-34ac/34bc-5p/449abc/449c-5p	1,000893	14	5	0,5877
miR-224	0,98261	12	4	0,1447
miR-143/1721/4770	0,968544	10	5	0,3427
miR-448/448-3p	0,95564	13	5	0,7232
miR-421	0,945048	10	5	0,4255
miR-154/872	0,932469	6	4	0,0654
miR-505/505-3p	0,917117	8	4	0,1145
miR-136	0,91024	7	5	0,3129
miR-499-5p	0,892029	8	5	0,4562

miR-221/222/222ab/1928	0,886575	9	5	0,5914
miR-544/544ab/544-3p	0,8852	10	5	0,7384
miR-376abd/376b-3p	0,872741	6	5	0,3585
miR-382	0,871178	6	5	0,3637
miR-384/384-3p	0,84581	7	5	0,5664
miR-219-5p/508/508-3p/4782-3p	0,836789	10	4	0,3647
miR-874	0,824123	6	5	0,561
miR-370	0,823955	7	5	0,6927
miR-28-5p/708/1407/1653/3139	0,820328	8	3	0,0883
miR-216b/216b-5p	0,817689	8	4	0,2861
miR-199ab-5p	0,817608	11	4	0,5365
miR-33ab/33-5p	0,815354	10	4	0,4443
miR-139-5p	0,810806	9	4	0,3758
miR-21/590-5p	0,80497	10	3	0,1546
miR-217	0,786108	6	5	0,7962
miR-504/4725-5p	0,783874	7	3	0,1002
miR-876-5p/3167	0,755992	8	3	0,1597
miR-196abc	0,749419	7	4	0,4352
miR-485-5p/1698/1703/1962	0,748479	8	4	0,5412
miR-378/422a/378bcdefhi	0,722647	5	4	0,3664
miR-299/299-3p/3563-3p	0,721936	4	3	0,0946
miR-490-3p	0,711318	5	4	0,4067
miR-542-3p	0,698332	6	4	0,5651
miR-197	0,683311	7	3	0,253
miR-296-3p	0,679308	4	2	0,0443
miR-361-5p	0,675471	5	4	0,5658
miR-338/338-3p	0,674872	6	4	0,7014
miR-375	0,661651	5	4	0,6426
miR-376c/741-5p	0,659742	5	4	0,654
miR-503	0,652739	6	4	0,86
miR-486-5p/3107	0,651463	5	3	0,2232
miR-208ab/208ab-3p	0,642794	4	4	0,6201
miR-10abc/10a-5p	0,636158	7	3	0,3906
miR-18ab/4735-3p	0,634969	7	3	0,3949
miR-487b	0,632151	2	2	0,045
miR-383	0,618383	5	3	0,3027
miR-339b/339-5p/3586-5p	0,593719	5	3	0,3799
miR-122/122a/1352	0,592865	5	3	0,3829
miR-346	0,578505	4	3	0,3545
miR-185/882/3473/4306/4644	0,567243	6	3	0,5977
miR-328a/328b-3p	0,558824	5	3	0,5239
miR-193/193b/193a-3p	0,553625	5	3	0,5496
miR-411	0,530048	3	3	0,4493
miR-146ac/146b-5p	0,505507	4	3	0,6944
miR-125a-3p/1554	0,504852	4	3	0,6986
miR-653	0,5038	4	3	0,7054

miR-192/215	0,495031	3	3	0,6203
miR-491-5p	0,488098	5	2	0,3178
miR-324-5p	0,442173	4	2	0,3935
miR-379/1193-5p/3529	0,424993	3	2	0,3739
miR-431	0,420305	4	2	0,4813
miR-875-5p	0,397015	3	2	0,4838
miR-126-3p	0,378773	2	1	0,1468
miR-551a	0,361428	1	1	0,1397
miR-451	0,254596	1	1	0,3737
miR-184	0,234521	1	1	0,4496
miR-191	0,182194	1	1	0,728
miR-134/3118	0,151202	1	1	0,9685
miR-99ab/100	0	0	0	1
miR-758	0	0	0	1
miR-615-3p	0	0	0	1
miR-496	0	0	0	1
miR-450a/451a	0	0	0	1
miR-210	0	0	0	1
miR-187	0	0	0	1
miR-127/127-3p	0	0	0	1

Supplementary Table S3 - BAV vs. TAV dilated aorta

miRNA	Score	Genes Targeted	Clusters Targeted	pval
miR-124/124ab/506	2,924743	152	8	0,0002
miR-23abc/23b-3p	2,75	114	8	<0.0001
miR-200bc/429/548a	2,480263	111	6	<0.0001
miR-1ab/206/613	2,434211	85	7	<0.0001
miR-181abcd/4262	2,275457	117	5	0,0003
miR-144	2,236842	93	5	<0.0001
miR-203	2,20234	81	8	0,0021
miR-15abc/16/16abc/195/322/424/497/1907	2,166147	115	6	0,0023
miR-145	2,144737	79	5	0,0001
miR-96/507/1271	2,12781	97	6	0,0011
miR-30abcdef/30abe-5p/384-5p	2,100767	115	6	0,0042
miR-300/381/539-3p	2,041504	89	6	0,0015
miR-182	2,002082	100	6	0,0042
miR-218/218a	1,999252	89	5	0,0007
miR-130ac/301ab/301b/301b-3p/454/721/4295/3666	1,910784	89	4	0,0005
miR-9/9ab	1,906525	108	5	0,0052
miR-340-5p	1,893878	116	6	0,03
miR-495/1192	1,889759	86	5	0,0016
miR-27abc/27a-3p	1,886339	99	7	0,0363
miR-320abcd/4429	1,857214	74	6	0,0033
miR-590-3p	1,851482	98	7	0,0471
miR-21/590-5p	1,724485	38	5	0,0004

miR-19ab	1,711269	96	6	0,048
miR-25/32/92abc/363/363-3p/367	1,688191	74	7	0,0495
miR-17/17-5p/20ab/20b-5p/93/106ab/427/518a-3p/519d	1,669565	100	5	0,0284
miR-204/204b/211	1,668883	68	4	0,0013
miR-150/5127	1,654146	34	5	0,0006
miR-543	1,61768	64	7	0,0517
miR-425/425-5p/489	1,571568	26	6	0,0025
miR-499-5p	1,567761	39	5	0,0018
miR-133abc	1,567064	62	5	0,0073
miR-28-5p/708/1407/1653/3139	1,565789	29	3	0
miR-205/205ab	1,542283	46	4	0,0011
miR-199ab-5p	1,540644	50	5	0,0045
miR-101/101ab	1,51436	73	5	0,0231
miR-377	1,5065	52	6	0,022
miR-488	1,499791	41	5	0,0038
miR-29abcd	1,494378	80	6	0,1342
miR-410/344de/344b-1-3p	1,491065	62	5	0,0147
miR-24/24ab/24-3p	1,48761	55	6	0,0314
miR-148ab-3p/152	1,476826	62	6	0,053
miR-135ab/135a-5p	1,446265	61	6	0,0661
miR-125a-5p/125b-5p/351/670/4319	1,431066	60	7	0,2263
miR-374ab	1,400382	56	6	0,0745
miR-137/137ab	1,398048	87	5	0,1575
miR-103a/107/107ab	1,386319	57	6	0,0901
miR-143/1721/4770	1,364122	37	6	0,0329
miR-142-3p	1,359672	40	3	0,0013
miR-128/128ab	1,34957	84	4	0,0649
miR-153	1,343822	61	5	0,0537
miR-539/539-5p	1,331745	57	5	0,0471
miR-186	1,328198	67	5	0,0892
miR-214/761/3619-5p	1,318567	58	5	0,0565
miR-33ab/33-5p	1,318399	41	5	0,0202
miR-155	1,304825	46	4	0,0098
miR-494	1,302884	54	4	0,0162
miR-221/222/222ab/1928	1,296563	41	5	0,0247
miR-339b/339-5p/3586-5p	1,275028	23	4	0,0032
miR-139-5p	1,262496	35	5	0,0235
miR-202-3p	1,25582	56	6	0,2821
let-7/98/4458/4500	1,254337	69	6	0,6287
miR-93/93a/105/106a/291a-3p/294/295/302abcde/372/373/428/519a/520be/520acd-3p/1378/1420ac	1,248438	61	6	0,4088
miR-421	1,240117	39	5	0,0368
miR-33a-3p/365/365-3p	1,224372	31	5	0,0262
miR-26ab/1297/4465	1,202886	65	5	0,2506
miR-141/200a	1,128875	56	5	0,2872

miR-22/22-3p	1,106175	48	3	0,0218
miR-132/212/212-3p	1,089013	36	5	0,1234
miR-329/329ab/362-3p	1,076311	31	4	0,0324
miR-433	1,075434	29	5	0,0915
miR-592/599	1,047327	21	5	0,073
miR-183	1,035625	39	3	0,0242
miR-544/544ab/544-3p	1,031026	41	5	0,285
miR-208ab/208ab-3p	1,030946	19	5	0,0752
miR-455-5p	1,023687	21	4	0,0287
miR-290-5p/292-5p/371-5p/293	1,014831	25	6	0,3968
miR-197	1,014677	24	4	0,0374
miR-31	1,00994	33	4	0,0674
miR-138/138ab	1,008904	48	3	0,0534
miR-873	1,006672	27	5	0,1527
miR-485-5p/1698/1703/1962	1,005986	33	4	0,0699
miR-338/338-3p	1,004288	30	4	0,0592
miR-216b/216b-5p	0,997792	25	5	0,1468
miR-361-5p	0,987721	21	5	0,1264
miR-411	0,971534	15	3	0,0102
miR-223	0,96721	29	4	0,0784
miR-224	0,966541	34	4	0,1068
miR-758	0,965225	21	5	0,1555
miR-149	0,958467	38	4	0,1466
miR-194	0,954289	30	5	0,2967
miR-7/7ab	0,93645	41	3	0,0681
miR-335/335-5p	0,932122	24	4	0,08
miR-376c/741-5p	0,923484	21	5	0,2284
miR-490-3p	0,917727	17	5	0,189
miR-448/448-3p	0,916221	49	4	0,4213
miR-382	0,911617	19	5	0,2257
miR-505/505-3p	0,911521	19	5	0,2259
miR-383	0,903483	15	5	0,1909
miR-376abd/376b-3p	0,893271	21	4	0,0954
miR-503	0,884781	32	4	0,2009
miR-193/193b/193a-3p	0,873932	21	4	0,114
miR-217	0,854446	29	4	0,2215
miR-196abc	0,849349	21	5	0,4521
miR-326/330/330-5p	0,8251	32	4	0,3481
miR-18ab/4735-3p	0,823166	23	4	0,2054
miR-375	0,819529	18	5	0,4961
miR-370	0,808627	23	5	0,7426
miR-384/384-3p	0,805158	21	5	0,6792
miR-190/190ab	0,78784	17	4	0,1977
miR-10abc/10a-5p	0,785288	27	2	0,0371
miR-431	0,779677	17	3	0,0674
miR-129-5p/129ab-5p	0,77266	36	4	0,719

miR-140/140-5p/876-3p/1244	0,769901	29	3	0,1526
miR-216a	0,742873	22	4	0,405
miR-34ac/34bc-5p/449abc/449c-5p	0,742304	46	3	0,5512
miR-219-5p/508/508-3p/4782-3p	0,738017	27	4	0,5734
miR-185/882/3473/4306/4644	0,731316	21	4	0,424
miR-126-3p	0,728714	6	2	0,0175
miR-496	0,728299	13	3	0,0849
miR-328a/328b-3p	0,72751	20	3	0,1307
miR-542-3p	0,690169	23	3	0,2211
miR-125a-3p/1554	0,688129	19	3	0,1768
miR-134/3118	0,680073	12	4	0,394
miR-136	0,669812	17	4	0,5863
miR-876-5p/3167	0,654859	20	3	0,2552
miR-874	0,646372	22	3	0,3115
miR-324-5p	0,627344	15	2	0,0768
miR-486-5p/3107	0,622916	13	3	0,2241
miR-378/422a/378bcdefhi	0,604286	11	4	0,7453
miR-346	0,601718	12	3	0,2564
miR-342-3p	0,579772	20	3	0,5096
miR-653	0,572104	10	4	0,9435
miR-146ac/146b-5p	0,57202	19	2	0,1629
miR-154/872	0,570101	11	3	0,3229
miR-487b	0,533247	3	2	0,0883
miR-122/122a/1352	0,528174	13	3	0,5363
miR-875-5p	0,520522	9	3	0,4516
miR-491-5p	0,470746	14	2	0,3058
miR-450a/451a	0,449763	2	2	0,1793
miR-296-3p	0,435898	6	2	0,2596
miR-379/1193-5p/3529	0,404006	8	2	0,3931
miR-191	0,384297	5	2	0,393
miR-504/4725-5p	0,379838	12	2	0,6258
miR-192/215	0,326426	8	2	0,8032
miR-184	0,324494	2	2	0,5684
miR-210	0,30519	2	2	0,679
miR-299/299-3p/3563-3p	0,277453	3	2	0,9314
miR-551a	0,228834	1	1	0,4083
miR-99ab/100	0,209101	4	1	0,5873
miR-615-3p	0,17929	1	1	0,6444
miR-451	0,157958	1	1	0,7843
miR-187	0	0	0	1
miR-127/127-3p	0	0	0	1
